# The Fixation Distance to the Stimulus Influences ERP Quality: An EEG and Eye Tracking N400 Study

**DOI:** 10.1371/journal.pone.0134339

**Published:** 2015-07-29

**Authors:** Estefanía Domínguez-Martínez, Eugenio Parise, Tommy Strandvall, Vincent M. Reid

**Affiliations:** 1 Department of Psychology, Lancaster University, Lancaster, United Kingdom; 2 Tobii AB, Danderyd, Sweden; Centre de Neuroscience Cognitive, FRANCE

## Abstract

In a typical visual Event Related Potential (ERP) study, the stimulus is presented centrally on the screen. Normally an ERP response will be measured provided that the participant directs their gaze towards the stimulus. The aim of this study was to assess how the N400 component of an ERP was affected when the stimulus was presented in the foveal, parafoveal or peripheral vision of the participant’s visual field. Utilizing stimuli that have previously produced an N400 response to action incongruities, the same stimuli sequences were presented at 0°, 4°, 8° and 12° of visual angle from a fixation location. In addition to the EEG data, eye tracking data were recorded to act as a fixation control method and to allow for eye artifact detection. The results show a significant N400 effect in the right parieto-temporal electrodes within the 0° visual angle condition. For the other conditions, the N400 effect was reduced (4°) or not present (8° and 12°). Our results suggest that the disappearance of the N400 effect with eccentricity is due to the fixation distance to the stimulus. However, variables like attentional allocation could have also had an impact on the results. This study highlights the importance of presenting a stimulus within the foveal vision of the participant in order to maximize ERP effects related to higher order cognitive processes.

## Introduction

When visual gaze remains relatively still, known as a fixation, the spatial resolution of the human visual field changes as a function of the distance from the center of the fixation point [1]. Researchers often divide the visual field into three areas: foveal, parafoveal and peripheral. Foveal vision is the area with the highest visual acuity, extending approximately 2° around the fixation point. In the parafoveal area the visual acuity decreases. It extends between 2° and 5° around the fixation point. The peripheral vision extends from 5° around the fixation point until the edge of the field of view. In this area, visual acuity decreases abruptly [2,3]. We usually move our eyes to place the fovea on the part of any object or location that we want to see clearly [4]. Parafoveal and peripheral vision also play a role when planning eye movements and extracting visual information (for a review see [5]). For example, it has been shown to be useful for gist recognition in natural scenes (e.g. [6–8]) and when examining familiar objects [9].

Usually during a fixation the attention is directed to the fovea. It is also possible to fixate on one location in space, yet to attend to other spatial positions in parafoveal or peripheral visual areas. These are known as overt and covert attention, respectively [10]. It is known that attention modulates the ERP waveform [11]. Thus the use of overt or covert attention will influence the ERP when a visual stimulus is presented to peripheral vision. In a visual experiment, covert attention usually occurs when the participant has been previously instructed to do so or has a cue that indicates that this should be done. In ERP studies of selective spatial attention, attended and unattended stimuli presented at different eccentricities have been found to differ in the magnitude of the early ERP components P1, posterior N1 and anterior N1, with all three having a larger amplitude for attended stimuli when compared with those that were non-attended [12–14].

The fixation distance to the stimulus, together with attention, alters the early components of an ERP. Little is known, however, about how advanced cognitive processes are altered when the stimulus is presented outside the foveal area. Advanced cognitive processes are related to the middle and late epoch periods of an ERP after stimulus onset [15]. One of these components is the N400, a widely investigated ERP component sensitive to semantic integration/violation in language research (for a review see [16]) and also exploited in action-related studies (for a review see [17]). This component can be observed as a negative deflection in the ERP waveform at frontal, parietal and temporal areas of the scalp—depending on the nature of the stimuli and task—at around 400 ms from the onset of the stimulus.

The main aim of the present study was to investigate whether the fixation location alters the cognitive processes that are generated by a visual stimulus. This was studied by analyzing the N400 response when participants were fixating at different distances from the stimulus location. We hypothesized that there would be a spatial distance from the stimuli where the N400 ERP component would start to become affected. This would cause a decrease in the amplitude of the N400, which could in turn affect final conclusions. These changes in the N400 component are expected based on the decrease of visual acuity with distance. To avoid changes in the ERP waveform due to differences in overt and covert attention, participants were asked to always attend to the stimulus. They were asked to maintain their fixation on a fixation cross that moved to different locations on the screen. They were also asked to attend to the stimulus without moving their eyes irrespective of where it appeared on the screen.

This study utilized stimuli that were validated in an earlier experiment conducted by Reid et al. 2009 [18]. In that experiment, simple sequences of actions were presented with two possible outcomes, one anticipated and one not anticipated. Results showed an N400 component in the unanticipated condition, whereas no N400 was observed in the anticipated condition. We aimed to replicate these results when presenting foveal stimuli. We also included three more conditions where the same stimuli were presented at 4°, 8° and 12° of visual angle from the participant’s fixation location. We calculated the averaged ERPs and compared the N400 component obtained in each of them with the foveal condition.

Gaze contingent stimulus presentation was used during ERP recordings to ensure that participants were directing their gaze to the right location on the screen at the beginning of each trial. We also used the eye tracking data during the ERP analysis to discard trials that contained eye movements or blinks. As a secondary aim of the study, we assessed the use of eye tracking data as a means to reject ERP trials that contain eye artifacts. We compared these results with the more normative detection of eye artifacts via the EEG amplitude information of the frontal electrodes [19]. We anticipated that the eye tracking data may be more sensitive to eye movement data than the algorithms related to eye artifact detection via EEG data.

## Materials and Methods

### Participants

Twenty-eight adult (13 females) aged from 18 to 49 years (m = 24.7, sd = 8.02) volunteered in the experiment. All participants were right handed and had normal or corrected-to-normal vision. All participants were given oral information about the experimental procedures and gave their written informed consent before participation. The study was approved by the Lancaster University Research Ethics Committee. For inclusion in the ERP grand average, participants were required to have at least 40 artifact-free trials per condition. A final data sample of eighteen participants was included in the analysis. The data of three participants had to be rejected because of technical problems during the experiment, one participant due to poor quality of eye tracking data and six participants because the minimum amount of artifact-free trials was not reached for one or more of the conditions.

### Stimuli

**Fig 1:**
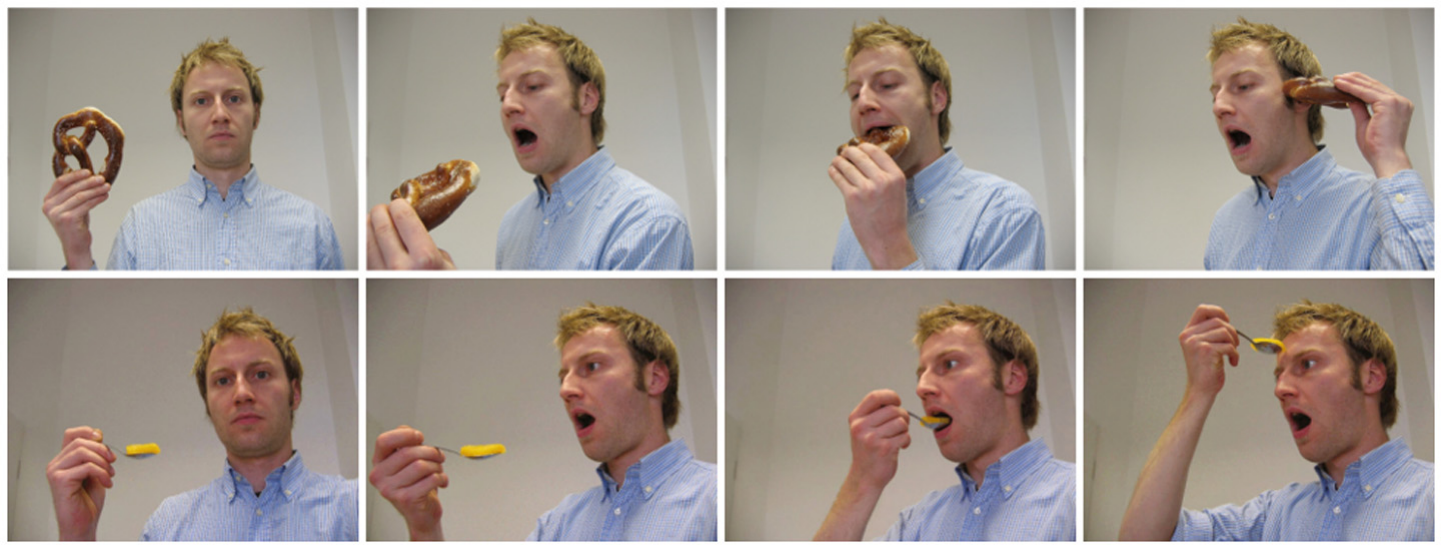
Action Sequence stimuli used in the study. Each row corresponds to a different stimuli set. First column: context of action; Second column: initiation of action; Third column: anticipated conclusion of action; Fourth column: unanticipated conclusion of action. The stimuli are the same as those used by Reid et al (2009) [18] in the adult study.

The stimuli were the same set of photographs used in Experiment 1 of Reid et al 2009 [18], which involved adult participants. Specifically, these were two sets of photographs depicting a male actor eating with a spoon or holding food. Each set consisted of three photographs. The first photograph displayed the general context of the action. The second displayed the initiation of the action. Each set of photographs finished with either an anticipated conclusion of the action with the spoon or food directed to the mouth, or, an unanticipated conclusion to the action was shown where the spoon proceeded to the forehead or food was positioned near the ear (Fig 1).

### Apparatus

During the experiment, both eye movements and EEG were recorded simultaneously.

#### Eye tracking

Eye movements were recorded at a sampling rate of 300Hz using a Tobii TX300 eye tracker (Tobii technology, Danderyd, Sweden). The eye tracking data were acquired using Tobii SDK 3.0 in Matlab (Mathworks, Natick, MA, USA) on the presentation computer.

#### EEG

EEG data were acquired by a 128-channel Sensor Net (Electrical Geodesic, Eugene, USA) with Net Station acquisition software running on a different computer. The EEG data were sampled at 250Hz. Electrode impedance was kept below 50KΩ. Raw EEG data were recorded with the vertex (Cz) as the online reference and re-referenced offline to an average reference.

#### Stimulus presentation

The stimuli were displayed on a 20-inch CRT monitor with a refresh rate of 60Hz. Psychtoolbox-3 for Matlab and custom made scripts were used for stimuli presentation [20,21].

### Procedure and experimental paradigm

Participants were seated at a fixed distance of 60cm from the eye tracker in accordance with the eye tracker requirements and in order to keep a similar field of vision across participants. First, the EEG net was positioned following the manufacturer instructions. When the electrodes’ impedance level was below the threshold, a five-point calibration routine of the eye tracker was performed followed by a validation of the calibration. The validation consisted of seven dots presented sequentially at different locations of the screen. Five of these locations corresponded with the potential locations of the fixation cross in the study. Before the experiment started, participants were instructed to fixate a fixation cross at the beginning of each trial and keep fixating on that location during the presentation of the stimuli. The fixation cross changed its location every trial and participants were asked to move their gaze to the fixation cross in order to start the next trial. The main purpose of changing the location of the fixation cross was to ensure that participants were engaged with the experimental design. Participants were also instructed to move as little as possible, to blink during the inter-stimulus interval and to pay attention to the stimuli without moving their eyes from the location of the fixation cross. The aim of the last instruction was to ensure that participants deliberately attended to the stimulus irrespective of the eccentricity of the image to the fixation location.

**Fig 2:**
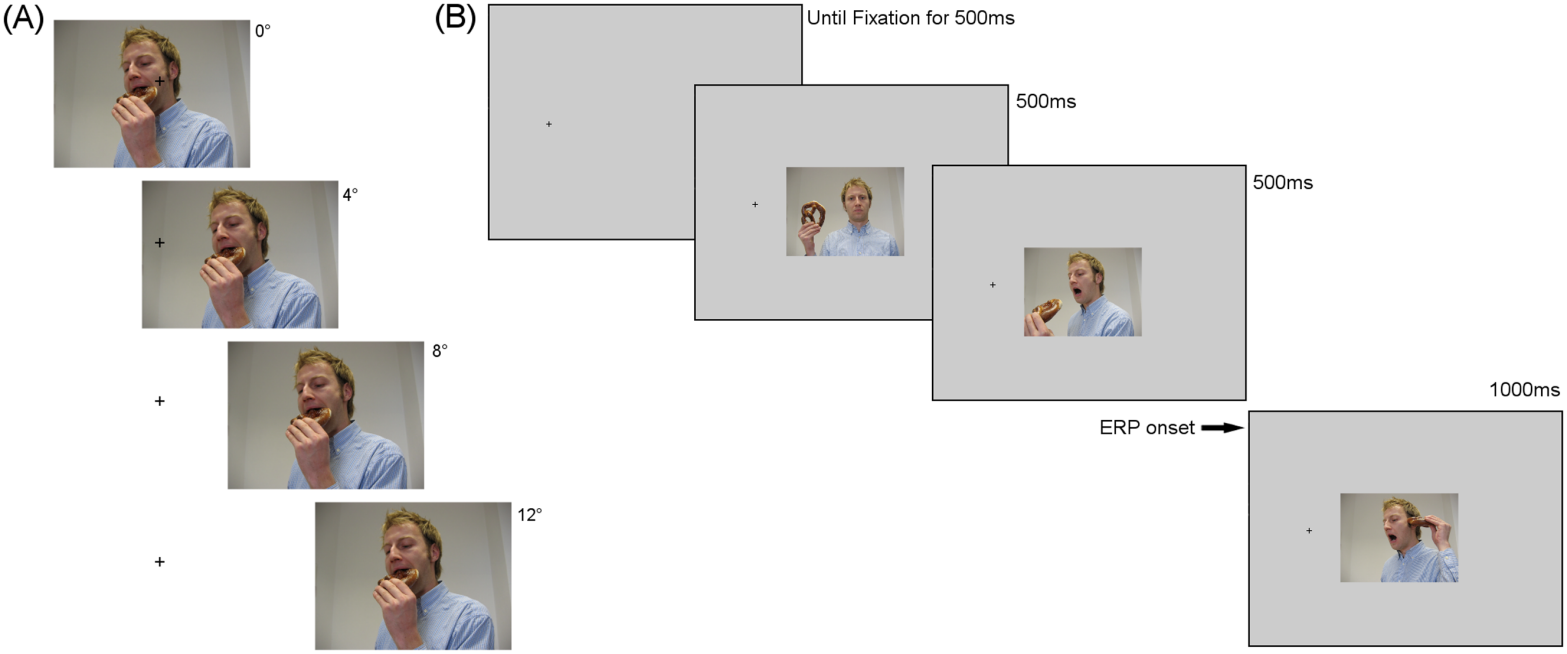
Location conditions and example of stimuli sequence. (A): The image appeared on the screen shifted horizontally depending on each specific location condition. (B): Example of a 4° condition trial. The stimuli sequence started by showing the cross on the corresponding location. Once the participant fixated it for 500ms, plus a random time between 200 and 400ms, the sequence of three images appeared on the screen. The cross remained on the screen during the trial. The ERP was time-locked to the presentation onset of the final image in the sequence.

Before each trial, a fixation cross of 0.4° of visual angle was presented on the screen. The position was selected randomly from five possible locations along the horizontal axis. The cross remained on the screen until the participant fixated it for at least 500ms. When this requirement was met, the trial started after a random period of 200–400ms. The three images were presented sequentially. The first two images were on the screen for 500ms each and the third image was on the screen for 1000ms. The stimuli were presented at a size of 10°x7.5° of visual angle. The experiment consisted of eight (2x4) different conditions: two levels corresponding to congruency (anticipated or unanticipated action) and four eccentricities corresponding to the distance of the image to the fixation cross (0°, 4°, 8° or 12° of visual angle). For the 0° condition, the mid-point of the main action in the anticipated and unanticipated third image was centered on the fixation cross. The main action for both congruent conditions was within the fovea of the participant when fixating the fixation cross. For the rest of the conditions, the stimuli were presented at the same vertical level, but the images depicting the action were horizontally shifted by the corresponding number of degrees of visual angle (Fig 2). The shifts were made randomly to the left or to the right for each trial and balanced by the end of the presentation. Each condition was presented 75 times, with a division of the two sets of stimuli of approximately half each. For each trial, the condition was pseudo-randomly chosen with the constraints that the same type of condition should not be presented three times consecutively.

The stimulus presentation was split into four segments of around 10 minutes each. Between each segment, a five-minute pause was given to the participants to rest, re-calibrate the eye tracker and rehydrate the EEG net when necessary.

### Data analysis

#### Eye tracking

Eye tracking data were analyzed using Matlab. First, a fixation filter was applied to detect fixations. The fixation filter was implemented based on the Tobii I-VT fixation filter [22,23], with a fixed velocity threshold of 30°/s for all the recordings. Interpolation of missing data, the merging of adjacent fixations and the discarding of short fixations were completed following the standard values suggested in the Tobii I-VT fixation filter description [22]. Eye tracking data were segmented into epochs that comprised 1200ms before the onset of the third image in the stimulus sequence and 1200ms following onset. Fixations were corrected every epoch using the validation data. The correction values were calculated independently for each cross location. For each validation location, the X and Y mean value of the samples collected was subtracted to the location of the validation dot. For every epoch, the X and Y values of the fixations were corrected using the correction value that corresponded with the trial location.

Eye tracking data were used to detect and reject the trials that did not meet the ERP analysis requirements. Trials were marked for rejection when: an eye movement larger than 1° was made during the ERP epoch, the participant’s point of gaze was not within the 1° around the fixation cross or a blink was detected. A trial was considered to contain a blink when there was a period of 75ms to 350ms of missing data preceded or followed by a saccade. Saccades before and after the blinks are considered eye tracking artifacts which are generated by the eyelid going up or down during a blink [24].

#### EEG data pre-processing

EEG data were analyzed using the Matlab toolbox EEGLAB v13.0.1 [25]. EEG data were first band-pass filtered between 0.3 and 30 Hz. The EEG recordings were segmented into epochs and baseline corrected. Each epoch comprised a 100ms baseline before the onset of the third image (namely from the last 100ms of the second image in the sequence) and 1000ms of the third image, displaying the final state of the action. Trials marked as rejected during the eye tracking analysis were automatically discarded. For the remaining trials, electrodes were marked as bad when the absolute amplitude exceeded 100μV. An electrode was marked as bad for the entire recording when it had been marked as bad for more than 40% of the trials. A trial was marked as bad and rejected when more than 10% of the electrodes were marked as bad during the trial. The remaining electrodes that were marked as bad for individual trials were interpolated. Electrodes marked as bad for the entire recording were completely interpolated. After the rejection of trials and interpolation of electrodes, the EEG data were re-referenced to the average reference and segmented into the eight conditions described above.

In order to evaluate the new technique for rejecting trials based on eye tracking data, we also conducted the same ERP analysis using the EEG analysis software Net Station. The EEG data analysis was the same as described above except for the trial rejection procedure. A standard artifact detection procedure, that is based on EEG amplitude, was used to detect eye movements and artifacts [19].

#### Measuring the ERP N400 effect

**Fig 3:**
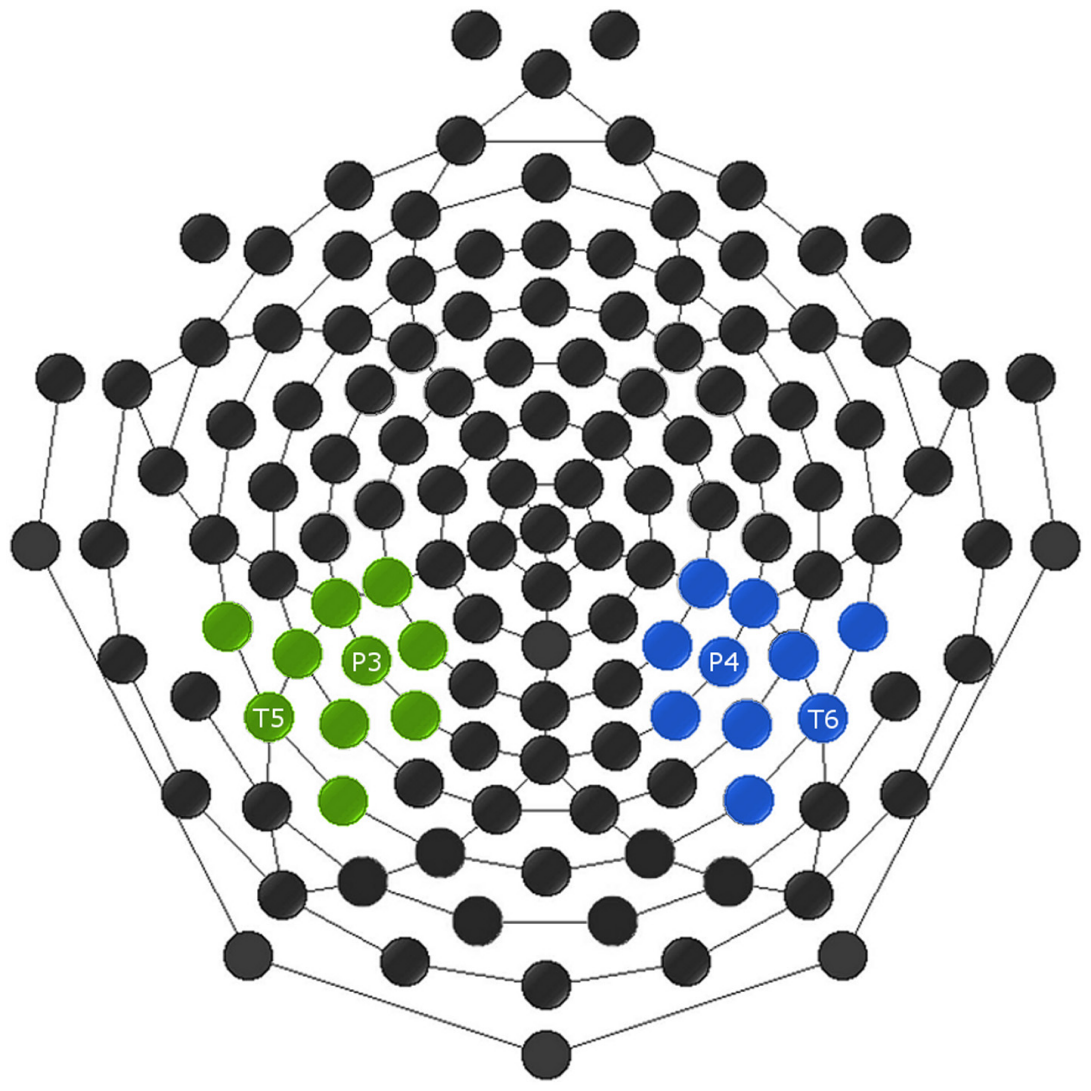
Clusters of electrodes selected for the analysis of the N400 component. A cluster of ten electrodes was selected based on where the N400 component was observed in the right parieto-temporal region of the scalp (blue). Symmetrically, a cluster with the corresponding electrodes was chosen in the left parieto-temporal region (green).

In the 0° condition, a clear negative peak was observed in the unanticipated condition in the right parieto-temporal area in the general time window expected for an N400 component, whereas this was not the case for the anticipated condition. In agreement with N400 literature [17,26], a time window was chosen around the amplitude peak of the N400 component in the right parieto-temporal area from 300–450ms after the stimulus onset. Due to a constant delay of EEG data inherent in the anti-aliasing filters of the GES 300 amplifier that was used, the data are offset by 36ms in all reported ERP waveforms and time windows. This information was communicated by EGI on August 29, 2014. A cluster of 10 electrodes including P4 and T6 where the N400 effect was observed were averaged together for each of the eight conditions. The corresponding electrodes in the left part of the scalp were also averaged together for each condition (Fig 3). In the area where the effect was observed, one condition displayed a defined peak, whereas the other did not. This effect had the same morphology as the one observed in the original study [18] for the adult data, and therefore the same window analysis technique of Hoorman et al. (1998) [27] was utilized. In this analysis, a repeated measures analysis of variance (ANOVA) is conducted with time as an additional within-subjects factor. A significant interaction of condition with time means that the waveform evolves differently for each condition and therefore the effects are different. Using time as a factor can be problematic in an ANOVA as adjacent sample points correlate more highly than more distant points. Although the conservative Greenhouse-Geisser method is intended to account for violation of sphericity, this method is also the standard approach used to deal with correlations between observations [27]. Thus, all the statistical results reported were corrected with the conservative Greenhouse-Geisser method.

To study whether the N400 component changed significantly between eccentricity conditions, we analyzed the differences in ERP amplitudes by a 4 x 2 x 2 x 13 general linear model repeated measures ANOVA analyzing eccentricity (0°, 4°, 8° and 12°), congruency (anticipated, unanticipated), hemisphere (parieto-temporal right and left area) and time (13 samples at one sample per 12ms). We were interested in the triple interaction effect between eccentricity, condition and hemisphere. Follow up analyses with the same factors but pairing between eccentricity conditions showed which eccentricity pairs had a significant difference in their N400 component. No effects with time in these analyses indicate that the evolution of the waveform is always similar within and between most of the conditions, even if the amplitudes are different and significant between eccentricities, hemisphere and congruency.

We also ran four independent statistical analyses to compare the results of the N400 differences between congruency conditions for each of the eccentricity conditions. Variances of ERP were analyzed by a 2 x 2 x 13 general linear model repeated measures ANOVA analyzing congruency (anticipated, unanticipated), hemisphere (parieto-temporal right and left area) and time (13 samples at one sample per 12ms). The aim of this approach was to apply the standard statistical methodology used to report results in ERP studies. This is, whether the component of interest is different between conditions. We were interested in the interaction effect between hemisphere and congruency. Follow up time window analyses showed which eccentricity and hemisphere had a significant difference in the N400 component between congruency conditions by the interaction of congruency and time.

In addition, the visual ERP component N1 (channels 71, 72, 75 and 76) was analyzed between eccentricity conditions. A repeated measures ANOVA analysis was conducted with the eccentricity condition as a within-subjects factor. The analysis was based on the average amplitude measured during the time window where the N1 peak was observed for each eccentricity condition. For this analysis, both congruency conditions were collapsed into a unique condition for each subject since both of them had a similar N1 visual component. All the analyses were conducted using IBM SPSS statistics 19.

## Results

The time window analysis that included the factors hemisphere (right and left), congruency (anticipated and unanticipated), eccentricity (0°, 4°, 8° and 12°) and time indicated the following significant effects: There were main effects of eccentricity, F_(2.41, 41.04)_ = 12.75, p < 0.001, and congruency, F_(1.0, 17.0)_ = 9.003, p = 0.008. There was an interaction effect of eccentricity and hemisphere, F_(2.64, 44.90)_ = 3.67, p = 0.022. More important for the hypothesis of the study, there was also an interaction effect of eccentricity, hemisphere and congruency, F_(2.06, 35.02)_ = 4.043, p = 0.025. Six follow up time window analyses including the same factors but pairing between eccentricity conditions showed that there were interaction effects of eccentricity, hemisphere and congruency only between the eccentricity pairings of 0°–8°, F_(1.0, 17.0)_ = 7.51, p = 0.014 and 0°–12°, F_(1.0,17.0)_ = 6.6, p = 0.02.

**Fig 4:**
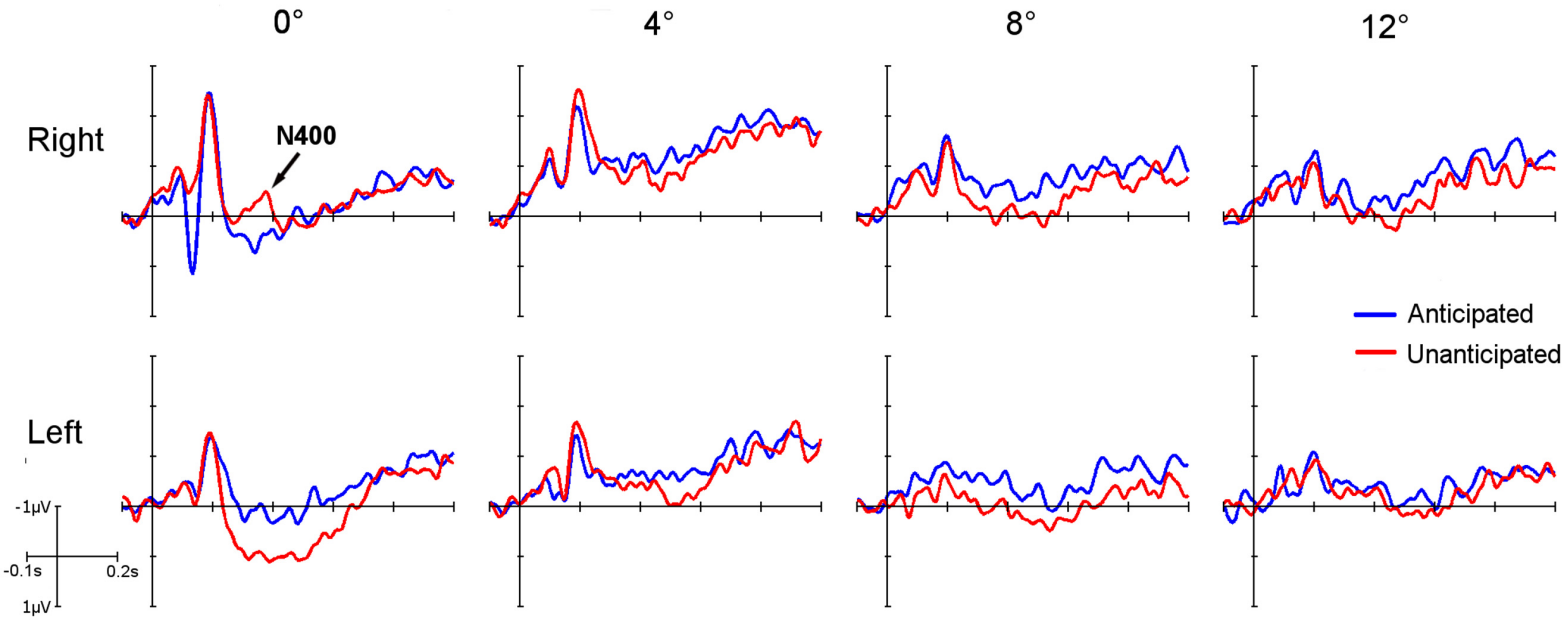
ERP averaged waveforms of the different location conditions in the parieto-temporal right and left clusters. The colors indicate the two different congruent conditions: anticipated (blue line) and unanticipated (red line). The N400 effect can be mainly observed in the anticipated condition for the 0° condition in the right parieto-temporal region of the scalp (arrow), and decreased in amplitude for the 4° condition.

Four individual statistical analyses, one for each of eccentricity condition, showed the effect that the fixation distance to the stimuli had on the N400 component of the ERPs. The 0° condition displayed an N400 component in the right parieto-temporal region of the scalp in the unanticipated condition but not in the anticipated condition. No N400 effect was observed in the left region (Fig 4 - 0°). For the 0° condition, a time window analysis including hemisphere, congruency and time indicated an interaction effect of hemisphere and congruency, F_(1.0, 17.0)_ = 6,245, p = 0.023. Two follow up time windows analyses were conducted, one for each hemisphere, including congruency and time as within-subject factors. In the right parieto-temporal region of the scalp, the results indicated a congruency by time interaction in the repeated measures ANOVA, F_(3.51, 59.77)_ = 2.838, p = 0.038. This means that the N400 component evolved significantly different in each congruency condition in the right region for the 0° condition. No effects were observed in the left parieto-temporal region of the scalp.

**Fig 5:**
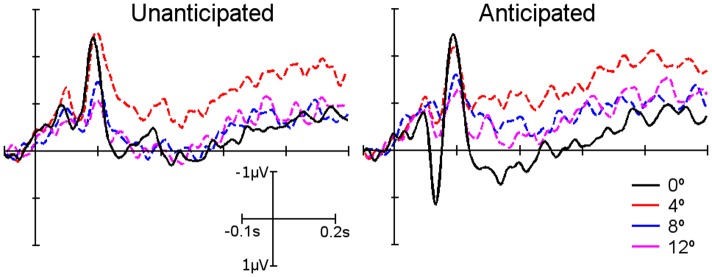
ERP averaged waveforms of the unanticipated condition (left) and anticipated condition (right) in the right parieto-temporal cluster. The different colors indicate the four different location conditions: 0° (solid black line), 4° (dashed red line), 8° (dashed blue line) and 12° (dashed magenta line). The N400 component can be observed in the unanticipated condition at 0° and decreased in amplitude at 4°.

For the 4°, 8° and 12° conditions, the ERP waveform decreased in amplitude between eccentricities for all the components across the scalp compared to the 0° condition. In the case of the N400 component, the effect showed a decrease in amplitude for the 4° condition and no N400 component was observed for the 8° and 12° conditions (Fig 5). Three more time window analyses were conducted, one for each of the remaining eccentricities (4°, 8° and 12°), including hemisphere, congruency and time. The results showed no interaction effects of hemisphere and congruency for any of the three eccentricities. Six individual time window analyses for the eccentricities 4°, 8° and 12° for left and right hemisphere of the scalp indicated no significant interactions: in the 4° condition, the congruency by time interaction ANOVA was not significant in the right parieto-temporal region, F_(4.03, 68.47)_ = 0.73, p = 0.575, as the N400 effect in the unanticipated condition was reduced in amplitude (Fig 4 – 4°). In the 8° and 12° conditions, the right parieto-temporal region of the scalp did not display an N400 effect in the unanticipated condition (Fig 4 – 8° and 12°). This was also shown in the congruency by time interaction in the ANOVA conducted for each of the two remaining eccentricities, where no significant differences were obtained in the right parieto-temporal region (F_(4.50, 76.38)_ = 0.496, p = 0.759 and F_(4.51, 76.77)_ = 0.948, p = 0.449, 8° and 12° respectively). No N400 effect was observed in the left parieto-temporal region for any of the eccentricities (Fig 4). This observation was confirmed by the results of the congruency by time interaction in the three repeated measures ANOVA analyses conducted, one for each eccentricity (4°, 8° and 12°).

The amplitude of the early visual components, including P100 and N100, decreased proportionally along the scalp, being reduced in amplitude as a function of the increase in visual degrees away from the displayed image. The highest mean amplitude of the measured visual N100 ERP component was observed when the stimulus was within foveal limits: 0° (-1.68μV, SE = 0.30). The mean amplitude of this component was reduced in amplitude when showing the stimulus within parafoveal limits: 4° (-1.09μV, SE = 0.31). The lowest mean amplitude was observed when the stimulus was presented within peripheral vision: 8° (-0.60μV, SE = 0.20) and 12° (-0.81μV, SE = 0.25). These differences were confirmed in the main effect of eccentricity in the repeated measures ANOVA of the visual component N100, F_(3, 51)_ = 7.12, p = 0.003. Analyses pairing between eccentricities showed significant effects between the 0° and 8° condition, F_(1, 17)_ = 17.21, p = 0.001 and 0° and 12° condition, F_(1, 17)_ = 6.67, p = 0.02. No significant effect was obtained between the pairing 0° and 4° condition.

### Comparison of artifact detection techniques

The same results were obtained when applying the statistical analyses to the EEG data that was pre-processed using a standard ERP procedure based on EEG amplitudes. A significant difference in the congruency by time interaction was shown in the ANOVA, F_(3.75, 63.884)_ = 2.82, p = 0.035 in the 0° condition for the right parieto-temporal region of the scalp. There were no differences in the left hemisphere in the 0° condition. Also, no differences were found for 4°, 8° and 12° conditions in parieto-temporal right and left regions.

## Discussion

The aim of this study was to investigate how the N400 component of an ERP is affected by visual eccentricity when images are presented as stimuli. For this purpose, we showed N400 inducing stimuli from Reid et al. (2009) [18] at different eccentricities from the fixation point of the participant. We expected to observe the same N400 effect in the 0° condition in our study as the participants’ foveal vision was within the critical element of the stimulus.

In the original study, an N400 component was observed in the unanticipated but not in the anticipated condition across the frontal, central and parietal regions of the scalp. We partially replicated these results, finding an N400 component with the same morphology in the unanticipated but not in the anticipated condition but with a moderately different scalp distribution. Our results show this effect across the right parieto-temporal region. There are at least two aspects related to the experimental design that could have influenced these spatial differences. First, there were technical differences between the studies as they employed different EEG systems. In the original study, the EEG system consisted of 23 electrodes located according to a 10–20 system. During the analysis, the data were re-referenced to the linked mastoid electrodes. In the present study, a high-density array of 128 electrodes was used. The linked mastoid reference is not ideal with high-density EEG systems. The recommended reference method is the average across all the electrodes since a good approximation of a zero potential of the head is obtained [28,29]. This aspect could have contributed to the differences observed as ERP waveforms are known to change in amplitude depending on the reference method applied [30,31]. However, a visual inspection of the grand average obtained after re-referencing the data to the linked mastoid revealed that the N400 effect did not changed substantially, having the same scalp distribution and morphology as with the average reference.

A second factor to explain the different scalp distribution of the N400 component, is the changes made to the paradigm with respect to the original study. Few changes were made in order to modify the paradigm to fit the research questions within the present study. Those changes that did exist comprised (a) higher number of times that the stimuli were presented to the participant, (b) the presence of the fixation cross also during the presentation of the stimuli and (c) the given task of fixating at the cross but attending to the stimuli irrespective of the location of the stimuli.

The N400 effect that is obtained during the viewing of images is commonly reported in frontal and central locations [17]. However, Shibata et al. (2009) [32] reported a parietal N400 effect when studying cooperative actions using pictures. Some authors also report a right lateralization of the N400 due to action images [33–35]. Moreover, the N400 effect found in the present study matches with the N400 effect usually found in language studies [16].

Regardless of the N400 distribution across the scalp, the most important result of the current study shows that this component was affected as soon as the main part of the stimulus was not within foveal vision. In the 4° condition, the fixation cross was still on the critical stimulus element within parafoveal limits and the N400 component could be observed in the unanticipated condition in the right parieto-temporal ERP cluster (Fig 4 - 4°). However, at 4° the N400 had decreased in amplitude, leading to a non-significant statistical result when applying the same time window analysis as the 0° condition. Additionally, the N400 effect appeared later in time, suggesting that the stimulus required more time to be processed. A shift in the time window in order to index the shifted N400 effect within the window in this condition did not lead to significant results. When the critical element of the stimulus was within the limits of peripheral vision (8° and 12° condition) no N400 effect for the unanticipated condition could be observed (Fig 4 – 8° and 12°). In these conditions, the fundamental aspect of the stimulus was still within visual field limits and therefore eccentricity clearly influenced the cognitive capacity to process this information.

Taking into account the results obtained in the present study regarding eccentricity, we hypothesized that the N400 component would be affected when computing an averaged ERP combining trials presented at various eccentricities, which might happen in canonical EEG studies not taking eye tracking information into account. We confirmed this hypothesis by means of a simulation analysis. We intermixed trials from across stimulus location conditions and applied the same N400 analysis to the new sets of ERPs. The N400 effect was consistently nullified when valid foveal trials were intermixed with at least 10% of the trials from other locations. The risk of intermixing trials from different eccentricities might not be high in adult ERP studies since fixating a specific part of the screen is an easy instruction to follow. Some experimental participants, however, are not able to follow instructions, for example, children and infants. This means that they may not fixate at the critical part of the stimulus on all trials, with the result that the stimulus is presented at a mix of eccentricities. As the results of the present study show, this will increase the risk of obtaining a diminished N400 effect, or even of nullifying the N400 differences between conditions.

To our knowledge, this is the first study that examines how the late components of an ERP are affected by the fixation distance to the stimuli. The current results show that there are effects on the N400 as a function of distance to the target location. The nature of the mechanisms that cause this to happen are still unknown. One candidate is spatial attention. Studies of spatial attention in word processing demonstrate that the N400 is modulated or even eliminated by selective spatial attention [36,37]. The design of the present study was intended to exclude the variable of spatial attention as participants were instructed to always attend to the stimulus, irrespective of its location. However, the lack of a more specific and demanding task during the experiment could may have influenced the participants’ allocation of attention.

The fixation distance to the stimuli has already been studied in relation with attention on early components of an ERP [11–14]. Our results are in line with previous findings, with a significant proportional decrease in the visual N1 component as the fixation distance to the stimuli increases. From the results obtained in this study, we can hypothesize that there is a relationship in terms of how the early and late components of an ERP are affected by the fixation distance to the stimuli. How different components interact is an area of research that requires further attention in the future.

The rate of exclusion was higher than it is usually reported in other ERP studies. Approximately 20% of the participants were excluded because they did not reach the minimum number of trials required for one or more of the conditions of the experiment. The main reason behind this was the nature of the task, with the presentation of the image outside foveal vision. Roughly 30% of the trials seen by the rejected participants were excluded because saccades were made towards the image when its location was not at zero degrees.

Eye tracking data were recorded simultaneously with EEG data. In addition to using eye tracking data to ensure that the participant fixated on the correct location at the beginning of each trial, the data were also used to detect trials that were contaminated with blinks and saccades. This approach was shown to be as valid as the traditional methodology based on assessing changes of the amplitude of the EEG data. However, the blink algorithm used was not fully robust, and visual inspection was required to detect false positives and false negatives. Some studies have used blink detection algorithms for different purposes (e.g. [38–41]). In these studies, the quality of the method used was not reported, most likely because no other physiological measurements, such as EOG, were compared with the obtained results. The present study suggests that in order to have reliable automatic blink information from eye tracking data, the improvement of blink detection algorithms would be necessary.

Having access to the participant’s gaze information during an ERP experiment has some advantages that can be used to ensure the quality of the ERP data. First, gaze-contingent techniques can be applied during the experiment to make sure that the participant fixates on the critical part of the stimulus. In our study, the fixation cross remained on the screen as long as the participant was not fixating on it for a minimum amount of time. This ensured that the participant was looking at the right location at the beginning of the trial and gave more control to the participant in terms of pacing the experiment. Additionally, eye tracking data can be used to discard invalid trials due to eye movements or blinks. This might not be necessary in studies with adult participants since the use of common detection techniques based on EEG amplitude are reliable. For more complex populations, such as infants, the selection of valid ERP trials is usually manually performed [42,43]. In this context, eye tracking could improve the quality of the final ERP waveform via utilizing the gaze data to help select those ERP trials where the infant attended to the stimuli.

In sum, we have shown that when viewing still images that induce N400 responses, the critical aspect of the stimulus needs to be within foveal vision. The inclusion of non-foveal trials that contain a reduced N400 effect can rapidly modify the ERP waveform, decreasing quality and potentially masking effects that have been generated by the stimulus. Our results suggest that the disappearance of advance cognitive components from the ERP response when a participant is not fixating within the main part of the stimulus is related with the fixation distance to the stimulus. Further research able to control covert attention would help understand if there are more variables involved in this process, like the allocation of attention in the stimulus.
